# Applications of CRISPR/Cas as a Toolbox for Hepatitis B Virus Detection and Therapeutics

**DOI:** 10.3390/v16101565

**Published:** 2024-10-02

**Authors:** Anuj Kumar, Emmanuel Combe, Léa Mougené, Fabien Zoulim, Barbara Testoni

**Affiliations:** 1 Cancer Research Center of Lyon, INSERM U1052, CNRS UMR 5286, 69008 Lyon, France; anuj.kumar@inserm.fr (A.K.); emmanuel.combe@inserm.fr (E.C.); lea.mougene@inserm.fr (L.M.); fabien.zoulim@inserm.fr (F.Z.); 2 The Lyon Hepatology Institute EVEREST, 69003 Lyon, France; 3 Hepatology Department, Hospices Civils de Lyon (HCL), Croix-Rousse Hospital, 69004 Lyon, France; 4 University of Lyon, UMR_S1052, UCBL, 69008 Lyon, France

**Keywords:** HBV, cccDNA, CRISPR/Cas, gene editing, diagnostics

## Abstract

Hepatitis B virus (HBV) infection remains a significant global health challenge, leading to chronic hepatitis, liver cirrhosis, and hepatocellular carcinoma (HCC). Covalently closed circular DNA (cccDNA) and integrated HBV DNA are pivotal in maintaining viral persistence. Recent advances in CRISPR/Cas technology offer innovative strategies to inhibit HBV by directly targeting both cccDNA and integrated HBV DNA or indirectly by degrading HBV RNAs or targeting host proteins. This review provides a comprehensive overview of the latest advancements in using CRISPR/Cas to inhibit HBV, with a special highlight on newer non-double-strand (non-DSB) break approaches. Beyond the canonical use of CRISPR/Cas for target inhibition, we discuss additional applications, including HBV diagnosis and developing models to understand cccDNA biology, highlighting the diverse use of this technology in the HBV field.

## 1. Introduction

### 1.1. HBV Life Cycle and Genomes

Hepatitis B virus (HBV) is a small, hepatotropic virus belonging to the *Hepadnaviridae* family. The infectious virions, also known as Dane particles, have a spherical, double-shelled enveloped structure with a 42-nm diameter. The envelope is made up of a cellular lipid bilayer and hepatitis B small-, middle-, and large-size surface antigens (S-HBsAg/M-HBsAg/L-HBsAg) surrounding a mature nucleocapsid formed by viral core proteins (HBc) and the genome. The genome is 3.2-kb relaxed circular DNA (rcDNA) with an incomplete plus strand and a complete minus strand and the viral polymerase (Pol) covalently linked to its 5′-end [1].

HBV binds to the sodium taurocholate co-transporting polypeptide (NTCP, encoded by the gene SLC10A1) to enter the host hepatocytes. The viral nucleocapsid is then delivered into the nucleus, where the covalently linked HBV Pol is removed from rcDNA, and the incomplete strand is repaired via host factors and chromatinized, leading to the formation of covalently closed circular DNA (cccDNA) [2].

**Figure 1:**
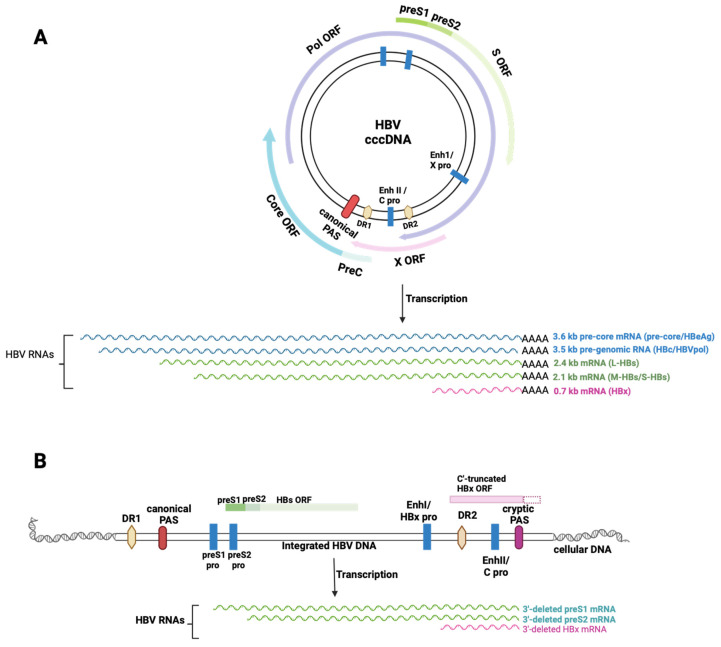
Schematic representation of the HBV transcripts and ORFs expressed from (**A**) cccDNA and from (**B**) the integrated HBV DNA. These HBV genomes are crucial for HBV chronicity, and they serve as targets for CRISPR/Cas9 approaches (pro: promoter; PreC: precore; DR: direct repeat; PAS: polyadenylation signal; Enh: enhancer) (adapted from [5,11,12,14]). Image created with BioRender.com.

The cccDNA is the sole template for the transcription of all HBV RNAs, which is regulated via several regulatory elements, including two enhancers, promoters (pre-S1, pre-S2/S, precore/core, and X), the epsilon encapsidation signal, and the polyadenylation signal (PAS) [3]. Transcription produces five main viral mRNAs, which are translated into seven proteins: (1) from the 3.5 kb pre-genomic RNA (pgRNA), HBcAg, and the reverse transcriptase/polymerase (Pol); (2) from the 3.6 kb precore mRNA, precore/ HBeAg; (3) from the 2.4 kb RNA, L-HBsAg; (4) from the 2.1 kb RNA, M-HBsAg, and S-HBsAg; (5) from the 0.7 kb RNA, HBx, a transcriptional activator [3,4,5] (Figure 1A).

The transcribed pgRNA is exported to the cytosol, and its 5′-end epsilon stem-loop structure binds to the HBV Pol [6]. This binding induces the packaging process, during which HBc self-assembles into an icosahedral capsid composed of 90 to 120 dimers and incorporates the pgRNA associated with Pol. Once encapsidated, the pgRNA serves as a substrate for reverse transcription via the HBV Pol, generating a new viral genome in the form of rcDNA [1]. Either the encapsidated rcDNA can be recycled back to the nucleus to replenish cccDNA, thereby maintaining the infectious pool, or the nucleocapsid can interact with the envelope proteins in the endoplasmic reticulum to assemble into mature virions. These virions are then released from host hepatocytes and infect neighboring hepatocytes [1].

The cccDNA serves as the episomal genomic reservoir for HBV and is highly resilient, with a half-life that can extend over several weeks or months. However, during hepatocyte division, it can be asymmetrically and randomly distributed between daughter cells. This uneven distribution results in variable amounts of cccDNA in these cells, leading to a gradual intrahepatic cccDNA loss over successive rounds of mitotic cell division [7]. A dramatic reduction in cccDNA levels was observed upon inducing mitosis in vitro in HBV-infected HepG2-NTCP and HepaRG-NTCP cells [8]. Similarly, the proliferation of HBV-infected primary human hepatocytes in vivo results in a drastic reduction in cccDNA in the liver of human chimeric mice [9].

In addition to cccDNA, the HBV genome is present in an integrated form intrahepatically [10]. This integrated HBV DNA can either be derived from the linear genomic DNA present in viral particles or produced from aberrantly processed rcDNA post-infection. The integrated HBV DNA lacks complete pol, precore, and core ORFs, and it is replication-incompetent. Transcripts generated from integrated HBV DNA are truncated at the 3′ end, and they terminate at a non-canonical PAS, known as a cryptic PAS, or use a downstream host PAS generating fusion transcripts. Despite being incomplete, these transcripts can still drive the expression of HBsAg and C-terminal truncated HBx protein [10,11,12] (Figure 1B).

Both forms of HBV DNA (cccDNA and integrated HBV DNA) play an important role in HBV chronicity (Figure 1). While cccDNA is primarily responsible for viral persistence, the integrated HBV DNA significantly contributes to HBsAg production, especially in HBeAg-negative patients [11]. HBsAg forms non-infectious subviral particles (SVPs), which are typically produced in much greater quantities than infectious virions. The excess SVPs lead to HBV-specific immune cell exhaustion and contribute to virus immunotolerance [13]. Around 5–50% of hepatocytes in chronic hepatitis B (CHB) or cirrhosis, and up to 90% hepatocytes in hepatocellular carcinoma (HCC) patients, harbor HBV integration. This observation suggests that viral DNA integration is pivotal in clonal hepatocyte expansion and hyperplasia, and ultimately in HCC development [11].

### 1.2. HBV Treatment and Cure

The currently approved nucleos(t)ide analogs (NAs) inhibit the reverse transcriptase activity of the HBV Pol, resulting in the decreased production of virions and the reduced recycling of viral nucleocapsids to the nucleus. Although this treatment is effective and is well tolerated in most patients, it requires life-long administration, as cessation often leads to viral relapse. Emerging treatments, including antisense oligonucleotides (ASOs), small interfering RNAs (siRNAs), and capsid assembly modulators (CAMs), target different aspects of the HBV life cycle. However, similar to NAs, they do not directly target cccDNA and integrated DNA, highlighting the unmet need for novel therapeutics that directly target these HBV genomes for an HBV cure [15,16].

The concept of an HBV cure is categorized into sterilizing, functional, and partial cures. A partial HBV cure involves HBeAg negativity with undetectable serum HBV DNA; however, because HBsAg remains positive, this type of cure is considered suboptimal. A sterilizing cure aims to eliminate intrahepatic cccDNA, as well as integrated HBV DNA, which is extremely challenging to achieve. A functional cure, characterized by a sustained loss of HBsAg for at least 6 months after the cessation of therapy, with or without seroconversion and undetectable serum HBV DNA, is considered optimal and the goal to strive for [16,17]. This indicates the situation achieved in patients who lose HBsAg spontaneously (acute or chronic infection) or after treatment. In contrast to a sterilizing cure, a functional cure allows the presence of cccDNA and integrated HBV DNA, but in their transcriptionally inactive forms. Understanding the mechanisms that regulate the transcription of HBV genomes (discussed in Section 2) is, thus, essential in developing novel and effective therapeutics in the aim of achieving a functional cure.

## 2. Regulation of HBV Transcription

The transcriptional activity of HBV DNA is regulated via various epigenetic modifications, including DNA methylation and histones post-translational modifications (PTMs), as well as host and viral proteins.

### 2.1. DNA Methylation

The methylation pattern of cccDNA is crucial in regulating HBV replication and pathogenesis. DNA methylation involves the addition of a methyl group to the fifth carbon of cytosine (5mC). This process commonly takes place within cytosine–phosphate–guanine (CpG) islands via host DNA methyltransferases (DNMTs), and it is associated with transcriptional silencing. Depending on the HBV genotype, the cccDNA contains two or three putative CpG islands located in the regulatory elements essential for viral gene expression [18,19,20].

The first CpG island, CpG I, overlaps the initiator ATG codon of the gene-encoding small HBsAg; CpG II overlaps both enhancers (Enh I and Enh II), the HBx promoter, and the core promoter, and CpG III covers the upstream region of the SPI promoter and initiator ATG codon of the polymerase gene [18,20]. While CpG I is less conserved and seldom methylated, CpG II and CpG III exist in all genotypes, and their methylation appears to be more conserved [18,19]. The methylation of CpG II and CpG III is associated with low levels of serum HBV DNA and HBsAg, respectively, in CHB patients [20]. Additionally, the methylation levels of CpG II were reported to be higher in liver biopsies of HBeAg-negative patients compared to those of HBeAg-positive patients [21]. Similarly, patients with occult HBV (negative for serum HBsAg) had higher methylation within CpG II than non-occult chronic patients, suggesting the possible link between HBV cccDNA methylation and HBV gene expression [22]. The gene-silencing impact of methylation has also been validated in HepG2-NTCP cells: transfection with in vitro synthesized rcccDNA containing methylated CpG II led to decreased levels of pgRNA compared to an unmethylated control [23]. Similarly, the cotransfection of an HBV DNA vector and a plasmid encoding a DNMT was associated with the inhibition of viral transcription [24]. Unexpectedly, Jain and colleagues reported non-CpG methylation in the CpG II of HBV genomes from liver tissues. Although non-CpG methylation was associated with transcriptional suppression in vitro, the significance of such epigenetic modification in liver pathogenesis requires further investigation [18,25].

Unlike cccDNA, only a few studies have focused on the methylation of integrated HBV DNA. It has been observed that the integrated HBV DNA sequences in SNU 398 cells, PLC/PRF/5 cells, and primary HCC tumors can be methylated [25,26]. A recent report demonstrated that the methylation level of integrated HBV DNA in PLC/PRF/5 cells is higher than that of cccDNA, and the transcription level of integrated HBV DNA negatively correlates with its methylation level. Since integrated viral DNA contributes to deleterious outcomes, the suppression of its expression may represent one of the mechanisms of host-cell self-defense [27].

Accordingly, the methylation of HBV genomes can lead to their transcriptional silencing and could thereby be conceived as a potential therapeutic approach to achieve a functional cure (discussed in Section 3.2.2).

### 2.2. Histone Modifications

Histone post-translational modifications (PTMs) are another crucial type of epigenetic modification that plays a vital role in regulating HBV gene expression. cccDNA is organized into a nucleosome-decorated minichromosome bound with histones 3 and 4 (H3 and H4), as well as non-histone proteins. cccDNA transcription has been shown to be regulated via the acetylation status of these histones in cell-culture models and in vivo in CHB patients [28]. While acetylation leads to active cccDNA transcription, the recruitment of histone deacetylase 1 (HDAC1) and hypoacetylation of H3 and H4 proteins is associated with reduced HBV replication and viremia in vitro and in vivo [28].

Using a cccDNA chromatin immunoprecipitation-sequencing (cccDNA-CHIP seq) approach, Tropberger et al. reported the first genome-wide maps of PTMs in the cccDNA minichromosome from de novo HBV-infected HepG2 cells, primary human hepatocytes (PHHs), and HBV-infected liver tissue. They showed the enrichment of active histone PTMs such as H3K4me3 and H3K27ac at HBV promoters [29]. In addition to acetylation, other PTMs have also been shown to be functionally linked to cccDNA transcription activity. For example, H3K122 succinylation leads to transcriptional activation, while H3K9me3 or H3K27me3 are associated with the repression of cccDNA transcription [30,31,32,33]. Locatelli et al. demonstrated the HIRA-mediated deposition of serine31 phosphorylated H3 variant H3.3 on transcriptionally active cccDNA in infected cells and human livers [34]. These observations suggest a tightly regulated epigenetic control of cccDNA expression.

Recently, Guan and colleagues demonstrated that the deposition pattern of histone PTMs on integrated HBV DNA in PLC/PRF/5 and on cccDNA from HBeAg-positive liver biopsies showed a high enrichment of the activating histone PTM H3K4me3 and a relatively low enrichment of the inhibitory histone PTM H3K9me3. This finding suggested that both integrated HBV DNA in PLC/PRF/5 cells and cccDNA from HBeAg-positive patients have higher transcriptional activity [27]. Further studies exploring other types of histone modifications and their impact on the transcription of integrated HBV DNA will be valuable. It is also essential to analyze the transcriptional regulation of HBV integration in PHHs under natural infection conditions and to better characterize integration using single-cell multi-omics technologies [27]. These results indicate that regulating epigenetic modifications of integrated HBV DNA could lead to transcriptional silencing and provide novel insights for improving the functional cure rate for CHB patients while reducing the incidence of liver cancer [27].

In addition to epigenetic modifications, cellular transcription factors and viral proteins, including HBx and HBc, play important roles in modulating cccDNA activity. Here, we briefly discuss the role of HBx in the context of HBV infection.

### 2.3. Role of HBx

Several independent studies utilizing different model systems (including cell-culture systems, the woodchuck model, and transgenic and humanized liver mice models) have unequivocally demonstrated the necessity of HBx to initiate cccDNA-driven transcription of the HBV RNAs and maintain virion production [18]. HBx is a multifunctional protein that employs various mechanisms to reshape the cccDNA minichromosome in favor of transcriptionally active chromatin [33], such as by influencing the recruitment of chromatin modifiers. Using a cccDNA-specific ChIP assay, HBx was shown to be recruited onto the cccDNA in HepG2 cells transfected with the HBV linear genome, and this recruitment parallels HBV replication and the association of histone acetyl transferases (HATs) [35]. In contrast, in cells expressing an HBx-defective HBV mutant, cccDNA is transcriptionally silent, and it is associated with decreased H3 acetylation and H3K4me3, increased repressive marks H3K9me2 and H3K9me3, and the increased recruitment of the histone deacetylases (hSirtl and HDAC1) [30,35]. While former study [35] highlighted the critical role of HBx, it is important to consider that this study was performed in HepG2 cells transfected with the linear HBV genome, and not in a model of natural infection.

To further demonstrate that HBx is essential for the transcriptional activity of cccDNA, some studies utilized HBV–HBx mutants competent for infection, but they observed that the cccDNA remains transcriptionally silenced. In host cells, this silencing is performed by the structural maintenance of chromosomes complex 5/6 (SMC5/6). However, HBx promotes the proteasome-mediated degradation of SMC5/6 by enabling the assembly of HBx-DDB1 (DNA damage-binding protein 1) and CRL4 (cullin 4 RING ubiquitin ligase) complexes, leading to its ubiquitylation [36,37]. The interplay between HBx and SMC5/6 is key to regulating HBV transcription. Recently, Yao et al. demonstrated that SMC5–SMC6 localization factor 2 (SLF2) interacts with the SMC5/6 complex to recruit cccDNA to PML bodies during HBV infection for viral transcription repression [38]. HBx antagonizes this phenomenon, leading to a disruption of the SMC5/6 complex without directly affecting SLF2 [38].

HBx also counteracts the recruitment of other HBV restriction factors, including SET domain bifurcated 1 (SETDB1) and high-mobility-group box-1 (HMGB1), to cccDNA, preventing its epigenetic silencing [30,33,39]. Apart from directly regulating HBV cccDNA transcription, HBx modulates a wide range of cellular signaling pathways associated with proliferation, apoptosis, autophagy, the cell cycle, epithelial–mesenchymal transition (EMT), metastasis, and metabolism, all of which contribute to the development of HCC [40]. In addition to full-length HBx, C-terminally truncated HBx produced from integrated HBV DNA has also been demonstrated to promote hepatocarcinogenesis [41,42]. Overall, targeting HBx could be a promising approach to inhibit HBV transcription and potentially prevent HBV-induced HCC.

In summarizing Section 1 and Section 2, we can conclude that there could be different approaches to inhibiting HBV, including directly targeting the HBV genomes or indirectly targeting host factors or viral RNAs involved in infection. Both approaches hold the potential to develop effective HBV-curative therapies.

This review describes the utility of CRISPR/Cas technology for these two approaches. Additionally, we also discuss the potential of CRISPR/Cas in other applications, such as HBV diagnosis and the development of models to study cccDNA biology.

## 3. Inhibiting HBV via the Direct Targeting of Viral Genomes through CRISPR/Cas

Due to simple design, extraordinary flexibility, and ease of use, CRISPR/Cas9 has gained widespread attention and is being explored as a promising strategy for targeting the genomes of several infectious viruses [43,44]. CRISPR was first described in 1987 as a series of repeated DNA sequences of unknown function and uncertain origin in *E. coli* [45,46]. Complexed with the effector protein Cas, the CRISPR/Cas was shown in 2012 to confer resistance to new incoming genetic elements, such as phages and plasmids, thereby providing adaptive immunity in bacteria [47].

Based on the composition of the effector complex, CRISPR/Cas systems are classified from Type I to Type VI systems. The Type II CRISPR/Cas system is a three-component complex consisting of the endonuclease Cas9, CRISPR RNA (crRNA), and transactivating CRISPR RNA (tracrRNA) [48,49]. The crRNA and tracrRNA may be combined via a linker loop sequence to create a single gRNA (sgRNA) without losing effector activity. The crRNA defines the specificity of the CRISPR/Cas complex to the target via nucleotide complementarity, upstream of the protospacer adjacent motif (PAM). This short sequence (3–8 bp long) is essential for the initial recognition of target DNA via Cas9. This recognition subsequently leads to the unwinding of the dsDNA sequence and the generation of an R-loop with an RNA–DNA pairing and an exposed ssDNA. In the prototypical CRISPR/Cas9 model, two endonucleolytic domains of Cas9 (RuvC and HNH) generate site-specific DNA double-strand breaks (DSBs) at the cleavage site located 3–4 bp upstream of the PAM. This DSB is detected via the error-prone, non-homologous end joining (NHEJ) repair mechanism, generating insertions or deletions (indels). Editing occurs at the expected site (“on-target”), or it may occur at significantly lower efficiency in non-targeted sequences sharing a close nucleotide similarity in the host genome (“off-target”).

This prototypical method has been extensively used in all research fields, including HBV research (Section 3.1), to create frameshifts, produce non-functional/truncated proteins, and promote target degradation. The CRISPR/Cas platform has also been actively engineered to improve on-target specificity and lower the off-target profile for therapeutic applications, to inactivate one or both endonucleolytic domains (generating nickase and dead Cas9) in order to prevent the DSB formation, to target the exposed ssDNA in the R-loop for deamination for base editing (base Editors), or to locally recruit transcriptional activator/repressors (CRISPRa and CRISPRi) and epigenetic editors (EEs) in order to precisely modulate the transcription and chromatin status, respectively (Section 3.2).

### 3.1. DSB-Based CRISPR/Cas

Several features of the HBV genome make it amenable to a CRISPR-based strategy. The compact size of the genome and the availability of several highly conserved regions facilitate the design of pan-genotypic gRNA candidates. Additionally, due to the overlapping nature of the genome, DSB cleavage resolved through indels could lead to additive effects on HBV replication inhibition. For instance, HBs ORF cleavage could potentially impact the Pol protein, targeting EnhII could affect HBx ORF, etc., thereby influencing multiple aspects of the virus life cycle. Using different HBV replication models, numerous studies have explored the potential of CRISPR/Cas9 to cleave HBV genomes. These studies have been summarized in the work of Martinez et al. and others [44,50,51]. gRNAs targeting highly conserved regions of the HBV genome were screened in easy-to-transfect HBV-producing stable cell lines. The effects of most effective gRNAs were then validated in more advanced and physiologically relevant cell-culture models, including HBV-infected HepG2-NTCP cells, primary human hepatocytes (PHHs), and in vivo in immunocompetent transgenic or immunodeficient humanized liver mice models. Targeted or broader off-target analyses were also performed in a few studies [50].

In this section, we briefly discuss recently emerged studies that (i) utilize CRISPR-Cas9 DSB-based technology to target HBV DNA in order to characterize the functional impact of HBV gene editing to better understand HBV biology and provide perspectives on building more efficient future therapeutic options (e.g., via gRNA multiplexing and a combination with NAs) and/or (ii) employ clinically relevant methods (e.g., extracellular vesicles (EVs) or lipid nanoparticles (LNPs)) to deliver “transiently expressed” CRISPR/cas9 components (e.g., mRNA-expressing gene editor or ribonucleoprotein (RNP) complex).

As mentioned in Section 2.3, due to its central role in cccDNA transcription and in promoting HBV-driven HCC, the HBx ORF remains a prime target for CRISPR/Cas9 targeting. The CRISPR/Cas9 targeting of the HBx gene in the HepG2.2.15 hepatoma cell line, which harbors the full-length integrated HBV genome, and in the HepG2 cells transfected with HBV-encoding plasmid, resulted in the inhibition of secreted HBsAg, decreased HBx mRNA and protein levels, and ultimately reduced cccDNA levels [52]. Targeting the HBx ORF reduced cell migration, chemotactic migration and invasion in both HepG2.2.15 and in Hep3B-transfected with HBV plasmid, highlighting a tumorigenic potential of HBx. Mechanistic insights confirmed the downregulation of several genes associated with the epithelial–mesenchymal transition (EMT) and cancer stemness (e.g., SNAIL1, TWIST1, ZEB1, and ZEB2, E-cadherin (CDH1), Vimentin, CD133, Thy1, CDH2, fibronectin, and alpha-SMA) upon HBx knockdown [52]. Transcriptomics/RNA sequencing data provided a comprehensive picture of alterations in genes regulating metabolism, histone modifications, viral infection, and signal transduction pathways. The impact of HBx targeting was further confirmed in 2D and 3D spheroid models, where the reduction was observed in number, size, and migration of spheroids, HBV mRNA expression, and cccDNA levels [52]. While gene regulation is a complex biological phenomenon, this work provides valuable insights into how HBx may alter the host transcriptome and how these changes might be reversed using CRISPR-Cas9 editing. It is noteworthy that effects of DSB cleavage at the same cleavage site in HBx region, as in this study, have also been investigated in other studies using different in vitro models, providing complementary data on HBV parameters [53,54].

Several virally and non-virally based delivery methods can be used to deliver CRISPR/Cas9 components into host cells [50,55]. Compared with plasmid or viral vector-based delivery, RNP-based delivery results in the short-term persistence of gene editing reagents, as RNPs are rapidly degraded via endogenous protease and nuclease enzymes. Due to their short lifespan, RNPs exhibits reduced off-target mutagenesis and, hence, are considered a superior method for delivering CRISPR/Cas9 components [56]. The RNP-based delivery of the Cas9 ortholog *Streptococcus thermophilus Cas9* (*StCas9*) and HBV-specific gRNAs has been utilized to target the cccDNA-like plasmid (rcccDNA) [57]. StCas9 recognizes a longer PAM sequence and offers distinct advantages [58,59]. As mentioned in Section 2.1, HBV cccDNA undergoes methylation; the authors utilized in vitro heavily methylated rcccDNA (meth-rcccDNA) and demonstrated, via an in vitro cleavage assay, that methylation may hamper gRNA efficiency. However, increasing the quantity of StCas9 restored cleavage efficiency, highlighting the importance of the gene-editor dosage in counteracting the negative effects of methylation. The authors also compared the effects of methylated and unmethylated rcccDNA upon transfecting them into HepG2-NTCP cells. Contrary to the in vitro results, similar reductions in unmethylated and methylated rcccDNA were observed, suggesting that DNA methylation does not impact the cleavage efficiency of StCas9 RNPs in cells [57]. 

The authors further investigated the role of rcDNA in HBV rebound. To study this, HBV-infected HepG2-NTCP cells were nucleofected with RNP at 7 days post-infection (dpi), and the effects on viral parameters were evaluated at 14 dpi/7 days post-nucleofection (dpn) and 24 dpi/17 dpn. Interestingly, HBsAg and cccDNA levels increased over time, indicating that HBV may rebound in RNP-treated samples. Notably, treatment with StCas9 alone did not result in a cccDNA decline at 7 dpn. It could be interesting to assess the outcome of on-target cleavage using StCas9 RNP (e.g., indels) in this naturally established cccDNA to determine the contribution of target mutations in the acquired phenotype. When combined with LAM, RNP hampered the rebound at 17 dpn and even reduced cccDNA levels, indicating that depleting the rcDNA pool before targeting cccDNA via Cas9 RNP could be beneficial for resolving HBV infection [57]. In contrast to previous work demonstrating cccDNA maintenance without requiring continuous replenishment [60], this observation suggests an important contribution of de novo synthesized rcDNA-to-cccDNA replenishment and viral rebound after CRISPR targeting in the HepG2-NTCP model. A further assessment of the effects of RNP + NA combination in primary liver cells and in vivo, as well as exploring off-targets, may advance the application of CRISPR/Cas9 therapy to NA-treated patients.

Further research on multiplexing gRNAs to increase the likelihood of priming cccDNA for degradation or to induce irreversible multiple-ORFs disruption has also been conducted [54,61]. Fei et al. advocate for using dual gRNAs to target HBV DNA utilizing the lentivirus delivery of Cas9 and gRNAs [54]. Ten gRNAs optimized for maximal conservation across genotypes A–H were screened for their anti-HBV efficacy in HepAD38- and rcccDNA-transfected HepG2 cells. The results demonstrated that these gRNAs could inhibit extracellular HBsAg and HBeAg, as well as intracellular 3.5 kbRNA, total HBV DNA, and cccDNA levels, with differential efficacy and without affecting cell viability. Pairing gRNAs targeting S/Pol, Pol/X promoter, or precore in a 2-by-2 multiplexing strategy achieved a synergistic reduction in viral parameters in HepAD38- and HBV-infected HepG2-NTCP cells [54].

Zhang et al. employed synthetic gRNA/Cas9 RNP to target pangenotypic HBV DNA sequences spanning overlapping regions (C/Pol, Pol/preS1, S/Pol, and Pol/HBx) [61]. Two gRNAs targeting Pol/S and Pol/HBx were selected from a screen in HepG2.2.15 for further validation. Both gRNAs, whether used individually or in combination, significantly suppressed cccDNA levels in stably transfected HepDE19 and HBV-infected HepG2-NTCP cell lines. MTT, Annexin V staining, and 7-ADD uptake experiments revealed no alterations in cell viability, apoptosis, or plasma-membrane integrity, respectively, indicating that gRNA/Cas9 RNPs are not toxic to human hepatoma cells [61]. Although this study provides a proof of concept that synthetic gRNA/Cas9 RNP can suppress HBV replication, a further in vivo evaluation is essential to assess long-term anti-HBV effects and immune-response analysis.

Extracellular vesicles (EVs), nanosized vesicles secreted from cells, have also been explored for therapeutic applications such as gene delivery [62]. However, cargo delivery via EVs may be limited due to low endosomal escape [63,64]. Zeng et al. addressed this challenge by developing a method to produce VSV-G coated extracellular vesicles (EVs) containing Cas9/gRNA RNP [65]. The engineered EVs successfully edited HBV DNA. An increased EVs payload was achieved by combining (i) a Cas9 fused to the Cryptochrome 2 (CRY2) protein and (ii) a membrane-anchored acylation tag fused to the CRY2–ligand–protein CIBN. The light-triggered heterodimerization of CRY2 with CIBN induces a rapid translocation of Cas9 to the membrane, promoting the encapsulation of gRNA/Cas9 RNP into EVs. Additionally, decorating the EVs with the fusogenic VSV-G protein decreased the endosomal entrapment via the recipient cells, enabling the nuclear delivery of the RNP in infected cells. The dual gRNAs-mediated cleavage of the HBV genome in vitro in an HBV-infected HepG2-NTCP cell line led to a reduction in extracellular HBeAg, HBsAg, HBV DNA, and intracellular HBcAg and cccDNA levels at 48 h post-EV incubation. In HBV transgenic and 1.2× HBV replicon (genotype C) replicating mice, a sharp decrease in HBsAg and HBeAg was observed at 7 dpi, along with reduced intrahepatic HBcAg staining [65]. Although this new delivery method shows promise by overcoming some of the challenges of Cas9 delivery and exhibiting low immunogenicity, extensive off-target analysis is crucial to ensure its safety for future clinical application. It is also important to recognize that the dual-gRNAs targeting of cccDNA may have limitations. For instance, studies have shown that the dual-gRNAs-induced cleavage of HBV plasmid [66], de novo established cccDNA in vitro [65,67], or HBV genotype C replicon in vivo [65] can excise HBV sequence (665–702 bp-length DNA sequences). However, these excised fragments can generate smaller, transcriptionally active CRISPR episomal variants, which may be relevant to consider in the context of an HBV cure [67].

Yi and colleagues conducted a proof-of-concept study using a clinically delivery-relevant method and cargo to target conserved regions of HBV genotypes A–D [68]. They utilized HuH7 cells transfected with in vitro synthesized cccDNA-like molecule and AAV-HBV1.04-transduced AML12 murine cells to identify the best gRNA combination for reducing HBV antigens (HBsAg and HBeAg). The most efficient combination, named g17 + g60, was tested in vivo using the SM-102 LNP delivery of Cas9-encoding mRNA for the transient expression of the Cas9 protein [68]. Notably, the dsDNA cleavage site of one of the lead gRNAs (g17) overlapped with a site identified in a previous study [69], highlighting the importance of this conserved region in regulating HBV replication.

The efficient bio-distribution and expression of LNP containing GFP-encoding mRNA in the liver of C57BL/6 mice were observed at 6 h post-injection, validating the SM-102 LNP delivery system. The delivery of Cas9 mRNA and HBV-targeting gRNAs (g17 + g60) effectively suppressed various viral parameters, including secreted HBsAg, HBeAg, HBV DNA, intrahepatic HBc and HBsAg, and cccDNA levels in infected tree shrews, without affecting intrahepatic immunostimulatory gene expression (IFNβ, IRF3, or ISG15) or ALT/AST levels. Additionally, a decrease in HBV RNA was observed in transgenic mice harboring 1.28-mer Gen A HBV integrated genome. The next-generation sequencing (NGS) of amplicons of selected candidate host genes (based on in silico predictions) in tree shrews and transgenic mice models revealed minimal off-target effects, suggesting that this approach may provide an opportunity to target cccDNA and integrated HBV DNA while reducing the risk of HCC. Interestingly, the reappearance of intrahepatic SMC5/6, an HBV restriction factor (as mentioned in Section 2.3), following HBx targeting could be beneficial, as it may promote the silencing of the potentially non-edited cccDNA molecules [68].

Though an increasing number of highly optimized Cas9 proteins and gRNAs have been developed for improved on-target and reduced off-target effects [70], prototypical Cas9 nuclease-based editing can still result in significant unintended outcomes. Off-target editing may cause unwanted genome alteration, including larger deletion/truncation, which can be difficult to monitor [71]. Moreover, the cleavage of integrated HBV DNA via prototypical Cas9 may generate DSBs in the host genome that may lead to genotoxicity through Chromothripsis—a phenomenon through which chromosomes shatter and extensively rearrange, resulting in complex genomic alterations [72]. Addressing these challenges may involve non-DSB-based genome editing, which circumvents the issue of DSB cleavage by employing alternative gene editing methods that do not involve creating double-strand breaks.

### 3.2. Non-DSB-Based CRISPR/Cas

#### 3.2.1. Base Editing

Thanks to the continuous evolution of the CRISPR/Cas9 technology and the recent development of base editors, it is now possible to introduce mutations in target DNA without causing DSBs. Two primary base editors can deaminate cytosine to thymine (cytosine base editor, CBE) or adenine to guanine (adenine base editor, ABE) [73,74]. As reviewed in Martinez et al. [50], Yang et al. first demonstrated the utility of CBE in targeting HBV cccDNA [75], followed by a study by Zhou et al., which showed the effect of CBE in inhibiting HBsAg expression from naturally integrated HBV DNA in PLC/PRF/5 cells [76]. Our recent research [77] not only confirmed these earlier observations [75,76] but also advanced the field of HBV base editing by providing the following novel insights:

(1) Effect on the established pool of cccDNA. Base editing should ideally be performed on an already established pool of cccDNA to mimic the situation observed in CHB patients. In our study, for the first time, the base editors were delivered after cccDNA establishment both in vitro and in vivo. We also demonstrated the administration of a base editor in lamivudine-pretreated hepatocytes showing (a) direct cccDNA editing in the context of a reduced rcDNA/cccDNA ratio, (b) the sustained inhibition of viral antigen production and replication with no rebound, and (c) the feasibility of combining BE and standard-of-care NA treatments.
(2) Assessing BE effects in HBV-infected primary human hepatocytes (PHHs). Our study evaluated the effect of BE side by side in both differentiated hepatocytes (HepG2-NTCP cells) and primary cells (PHHs), providing a more comprehensive in vitro assessment.
(3) mRNA-based delivery and in vivo data. We utilized mRNA-based delivery to allow the transient expression of BE in vitro, thus avoiding potential deleterious effects of lentiviral-based expression of BE. Furthermore, for the first time, we demonstrated the in vivo delivery of HBV-targeting base editing reagents using lipid nanoparticles (LNPs), displaying a therapeutically relevant strategy for systemic delivery to the liver. Our results unequivocally demonstrate the durability of the editing effect on cccDNA and, consequently, on viral replication and antigen production in vivo.
(4) Combining gRNAs and the use of next-generation BEs. We employed a combination of the two different gRNAs with a CBE-encoding mRNA, resulting in a reduction in all tested viral markers. In addition to using a standard BE4 base editor, we used two recently evolved cytosine base editors, PpAPOBEC1 [78] and CBE-T [79], demonstrating the broad applicability of the different base editors for reducing HBV markers.
(5) Off-target assessment. An extensive off-target assessment for the lead gRNAs included the screening of several hundred potential off-target sites as performed using RNase H-dependent amplification and sequencing (rhAmpSeq) [80]. We also confirmed that, in the context of HBV infection, next-generation CBEs display minimized off-target profiles.

Collectively, all three studies [75,76,77] provided valuable information for the further development of base editing as a promising approach to CHB treatment.

#### 3.2.2. Epigenetic Editing

As introduced in Section 2.1 and Section 2.2, both cccDNA and integrated HBV DNA transcription are regulated at the epigenetic level, involving processes such as methylation and histone PTMs. These regulatory mechanisms could be targeted via another CRISPR non-DSB strategy, epigenetics editors (EEs). These editors consist of a dCas9 fused to an effector protein that modulates transcription by epigenetically modifying the target DNA [81,82]. This approach may offer the specific, efficacious, and sustained silencing of viral gene expression, with the potential for a functional cure. Importantly, EEs do so without introducing the unintended chromosomal effects associated with classical gene editing approaches, including the CRISPR/Cas9 nuclease-based strategy. Since last year, two unpublished anti-HBV EEs programs have been disclosed, indicating growing interest in this approach.

At the International HBV meeting of 2023 and EASL congress of 2024, Chroma medicine Inc, a genomic medicine company, presented data on epigenetic repressors that could silence HBV markers by methylating DNA sequences with a high degree of conservation, including CpG islands [83,84]. A strong reduction in HBV antigens, HBsAg, and HBeAg was observed in HBV-infected PHHs. The epigenetic repression of HBsAg was maintained in HepG2.2.15 cells for nearly a month. Furthermore, a single administration of LNPs-encapsulating mRNA-encoding EE and specific gRNA in vivo led to a sustained reduction in HBsAg, with up to a 3.3 log and a 2.1 log reduction in HBsAg in AAV-HBV and HBV transgenic mouse models at 6 months after treatment. Additionally, through a tiered approach to assessing off-targets, the authors showed that these EEs are highly specific and do not induce off-target effects on host-gene methylation or changes in gene expression patterns in PHHs [83,84].

Similarly, another epigenome editing company, TUNE therapeutics, shared its data at the HepDART hepatology conference of 2023 [85]. The TUNE-401 EE, using a single gRNA, increases CpG methylation in the CpG II island. As mentioned above in Section 2.1, CpG II is a key regulatory element present in both HBV cccDNA and integrated HBV DNA. This methylation led to the suppression of HBV transcription in different in vitro and in vivo models. A near-complete suppression of viral DNA in PHHs in vitro, with durability beyond 550 days and more than 275 cell doublings, was observed. Similarly, high levels of repression were demonstrated in epi-edited cells in vivo in an HBV-infected FRG chimeric-liver mouse model upon the LNP-based delivery of an mRNA encoding episilencer and single gRNA [85].

Since HBV does not naturally infect non-human primates (NHPs), the authors utilized the PCSK9 gene as a proxy model to test their LNP-based platform in NHPs. They showed that a single dose of the PCSK9 surrogate episilencer was well tolerated by the animals, resulting in a 75% reduction in PCSK9 protein in serum and a 56% reduction in LDL-C levels. The authors believe this effect is durable, as the reduction in PCSK9 and LDL-C remained robust for one year, with methylation patterns staying unchanged over the same period. Although these preliminary data are promising, they need to be validated. Additionally, it would be interesting to get more insights on silenced HBV genomes and know whether we observe the same pattern of histone PTMs as seen in HBeAg-negative patients. Nevertheless, the aforementioned observations highlight the potential of EEs to achieve HBV silencing, paving the way for innovative treatments that could potentially offer a functional HBV cure.

## 4. Inhibiting HBV by Targeting Factors Other than HBV Genomes

### 4.1. Targeting HBV RNAs by CRISPR/Cas13

Apart from the widely used Cas9, McCoullough et al. have recently reported the utility of the Type VI RNA guided nuclease Cas 13b against HBV [86]. Cas13 offers notable advantages over Cas9, including the absence of any targeting sequence constraint (such as Cas9 PAM), and a negligible collateral RNA cleavage “off-target”, expanding the CRISPR toolbox to precisely target HBV RNA sequences. The authors demonstrated that CRISPR-PspCas13b efficiently reduces HBV RNAs, which, in turn, suppresses HBV DNA replication and viral antigen expression in different cell-culture models. They also observed a 50% decrease in serum HBsAg expression in a mouse model one week after a hydrodynamic co-injection of HBV plasmid (genotype A2), PspCas13b, and specific crRNA [86].

### 4.2. Targeting of Host Proteins via CRISPR/Cas

In addition to the direct targeting of HBV genomes, the non-DSB approach can also be utilized to indirectly target host-cell proteins influencing HBV replication, such as by utilizing CRISPR activation (CRISPRa). CRISPRa consists of a dCas9 protein fused to a transcriptional activator, like the catalytic subunit of p300 acetyltransferase, a universal regulator of gene transcription. An sgRNA can direct dCas9-p300 to host-gene regulatory elements, leading to transcription activation [87].

One such strategy includes the CRISPRa of host APOBEC/AID genes. The overexpression of APOBEC/AID strongly reduces HBV replication by introducing hypermutations; however, their long-term or uncontrolled expression could also induce the off-site deamination of the host genes [88,89]. To overcome this limitation, Kostyushev et al. leveraged the short-lived RNP-based delivery of CRISPRa containing dSaCas9-p300 and systematically attenuated sgRNAs (att-sgRNAs, with mismatches at specific positions) for the transient activation of APOBEC/AID genes. The authors demonstrated that this approach could mitigate the toxicity and pro-mutagenic effects of APOBEC/AID while retaining their potent anti-HBV activity. This study represents the first comprehensive assessment of the effects of “physiologically” overexpressed APOBEC/AID on HBV replication, as well as on cellular and genomic toxicity [90].

Qian et al. performed in vivo CRISPRa (dCas9-VP64-based) screening to identify genes involved in HBV integration hepatocarcinogenesis. They identified TONSL and TMEM65 as novel drivers promoting tumorigenesis. They demonstrated that both *Tonsl*-activated and *Tmem65*-activated tumors in immunosuppressed mice displayed histological and pathological features typical of malignant HCC [91]. These advancements in CRISPRa technology highlight the potential for novel therapeutic strategies targeting host factors involved in HBV infection and its associated complications.

In addition to its potential to inactivate HBV genomes or targeting viral RNAs or host factors, CRISPR/Cas has also been explored in other applications discussed in Section 6 and Section 7.

## 5. Delivery of CRISPR/Cas

Choosing the appropriate delivery method to deliver CRISPR/Cas9 components is crucial not only for the success of gene editing but also to ensure safety. An efficient delivery should allow the editing reagents to reach the liver without causing off-targets or other undesirable effects. The most robust approaches to the liver-targeted delivery of gene editors rely on viruses such as AAVs [55]. While these approaches offer certain advantages, they are associated with unwanted outcomes due to the prolonged expression of gene editors (Table 1). To overcome these drawbacks, several alternative non-viral strategies, including LNPs, nanoblades, and eVLPs, have been explored. These newer strategies enable the transient expression of gene editors, minimizing the risk of side effects (Table 1).

In the context of the HBV field, as summarized by Martinez et al. [50], several earlier studies have utilized viral vector-based approaches to deliver CRISPR/Cas9 for HBV inhibition. However, due to their limitations, the recent research trend has shifted towards non-virally based methods such as LNPs or EVs. These studies are highlighted in Table 2.

## 6. CRISPR/Cas for HBV Detection and Diagnosis

**Figure 2:**
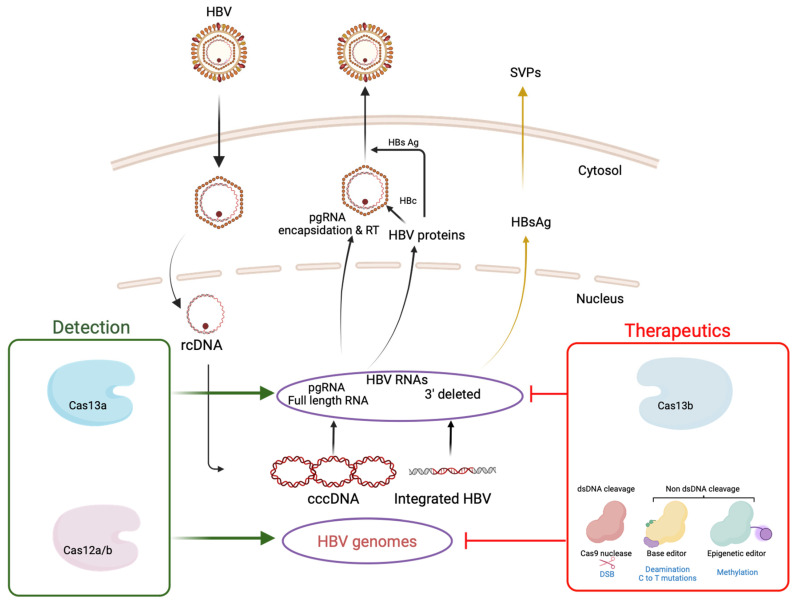
CRISPR/Cas-based approaches offer potential for both detecting and targeting cccDNA and integrated HBV DNA, which play crucial role in HBV chronicity. While Cas9 nuclease, base editors, and epigenetic editors can directly target viral genomes, Cas13b functions by targeting HBV RNAs. For detection purposes, Cas12 and Cas13 can be employed to detect HBV DNA or RNA, respectively. Although this figure primarily focuses on cccDNA and integrated DNA, Cas12 can also detect other HBV DNA species, including rcDNA. Image created with BioRender.com.

The early, fast, and accurate diagnosis of a pathogen is crucial for assessing disease progression and planning antiviral treatments. Various methods, such as antigen–antibody-based ELISA, PCR, or nucleic acid sequencing, are utilized for viral detection (Table 3). Due to robustness and high sensitivity, PCR is considered the gold standard for nucleic acid-based diagnostics. However, the cost of PCR reagents is high, and the technique needs sophisticated equipment and trained personnel. This limits its practical utility, particularly for point-of-care testing in high-disease-burden regions and resource-limited settings [98]. Therefore, there is a need for user-friendly technologies that can rapidly amplify nucleic acids while maintaining diagnostic accuracy, including single-nucleotide discrimination. Recently developed CRISPR-based diagnostics, utilizing isothermal amplification methods such as LAMP, SDA, MCDA, RAA, and Cas proteins (Cas12 or Cas13), show promise in meeting these needs. Unlike Cas9 (Type II)-based systems, Cas12 (Type V)- or Cas13 (Type VI)-based systems have the ability to trigger non-specific collateral trans-cleavage upon target recognition [98]. Cas12 detects dsDNA in *cis*-conformation and cleaves ssDNA collaterally, whereas Cas13 exhibits the collateral cleavage of ssRNA upon ssRNA detection in *cis*-conformation [99]. In the last few years, several research groups have developed Cas12- or Cas13-based diagnostics to detect HBV (Table 4). This highlights the potential of CRISPR/Cas toolbox components for both detecting and targeting HBV genomes using approaches outlined earlier (Figure 2).

Wang et al. and Zhang et al. demonstrated HBV detection with an LOD of 1 copy/µL using a CRISPR/Cas13a-based fluorescence detection assay [100,105]. Similarly, Ding et al. reported an LOD of 1 copy/µL using a CRISPR Cas12a fluorescence detection-based assay [101]. However, these assays were “one-to-one” signal output types, which could have limited their sensitivity. To enhance the sensitivity of the system, Bingxin Liu et al. proposed a CRISPR/Cas12a-responsive 3D magnetic DNA machine characterized by a “one-to-many” signal-output strategy [109]. This proposed 3D magnetic DNA machine was generated by cross-linking biotin-modified “seaweed-like” dendrimer DNA with streptavidin-coupled magnetic beads. The presence of HBV DNA in the serum initiates RCA, generating DNA nanowires with repeated sequence, which activates the CRISPR/Cas12a system multiple times. The trans-cleavage of the “seaweed root” via Cas12a results in the release of massive DNA duplex-enriched dendrimer DNA that, upon intercalation with Sybr Green 1 dye, generates a “one-to-many” fluorescence signal output [109].

In addition to Cas12a, a Cas12b-fluorescence-based HBV detection assay has also been developed [103,113]. Cas12b, a type V–B endonuclease, possesses DNase activity similar to that of Cas12a. As pre-amplification and detection in two different steps could be practically inconvenient and may increase the chances of false positive or false negative results, a system integrating LAMP amplification and Cas12b-based trans-cleavage/fluorescence detection in one pot termed HOLMESv2 was developed to detect nucleic acid in a one-step approach [114]. Haipo Xu et al. proposed HBV HOLMESv2 for the simple, quick, and specific detection of HBV using extracted genome or heat-inactivated clinical serum samples. When tested on 236 clinical samples, HBV HOLMESv2 demonstrated a specificity of 100% and a sensitivity of 99% compared to real-time qPCR [113].

**Figure 3:**
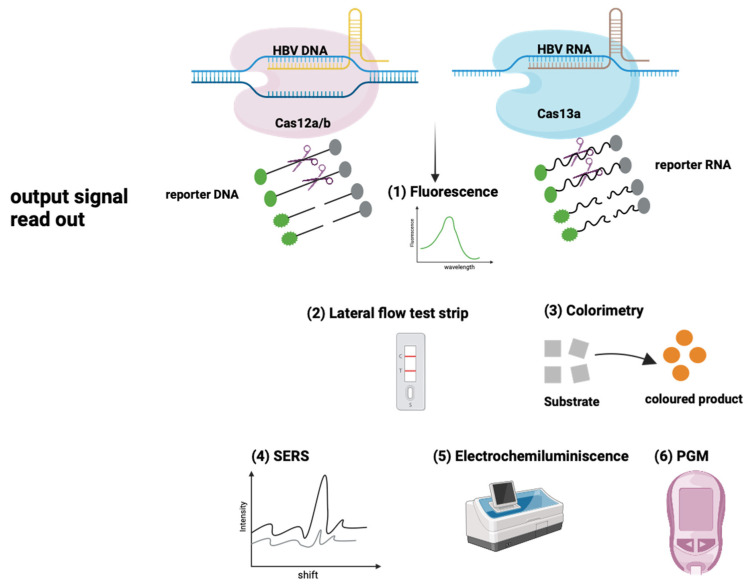
Detection of HBV using Cas12 and Cas13 can be achieved through various readout methods, including fluorescence detection, a lateral-flow immunochromatographic paper-strip assay, electrochemiluminescence, colorimetry, surface-enhanced Raman spectroscopy (SERS), and a personal glucose meter (PGM) (adapted from [98,112,115]). Image created with BioRender.com.

Due to the need for an expensive fluorescent reader, a fluorescence-based readout may be impractical for POC testing. Therefore, a lateral flow reading (LFA) method based on an immunochromatographic assay performed on a paper strip or colorimetric methods have been developed with the potential to allow HBV POC testing [101,103,104,106] (Figure 3). Tian Yuan et al. developed a rapid and portable RAA-CRISPR/Cas13a-based strip detection method that covers eight HBV genotypes. Importantly, they demonstrated that this method has great predictive value for detecting HBV DNA in patients with low viral loads and in those receiving antiviral therapy [111].

A study by Tian Li et al. demonstrated a universal strategy based on CRISPR/Cas12a and SDA to detect nucleic acid with multiple signal readouts (such as fluorescence mode, PGM mode, and LFA mode) [112] (Figure 3). The first step involves the strand-displacement amplification of the target sequence, generating a large amount of amplified DNA. This amplified DNA can partially hybridize with the template DNA to form a trigger DNA and activate the trans-cleavage activity of CRISPR-Cas12a, non-specifically cleaving ssDNA reporters and thereby completing the second recognition and signal amplification. Interestingly, the template DNA consists of two parts: a variable sequence for target recognition and a fixed sequence for composing trigger DNA. By simply changing the variable sequence, the gRNA scaffold can be adapted to probe the duplex trigger DNA output generated from different targets. The authors showed that this strategy could selectively identify HBV DNA and distinguish targets with single base mismatches.

Most CRISPR/Cas-based detection methods rely on pre-amplification, which may involve additional complicated steps and risks of false positive signals. To address this challenge, Choi et al. and Yuwan Du et al. reported the development of “amplification-free” nanobiosensing platforms for HBV detection via surface-enhanced Raman spectroscopy (SERS) [102,108]. They demonstrated that these methods could detect HBV at levels as low as 1 aM [102] and 0.1 pM [108], respectively.

## 7. Developing Models to Study cccDNA Biology

CRISPR technology has also been harnessed to develop new models for studying cccDNA biology by combining CRISPR-tagging and recombinant HBV minicircle technology [116,117]. Ding et al. devised an HBV rcccDNA-CRISPR tag-tracking system to study the spatiotemporal characteristics of the HBV minichromosome [118]. For the live-cell tracking of cccDNA, they established a cell line that constitutively expresses (i) a dCas9 fused to a split 14xGFP11 protein and (ii) the GFP1-10-NLS. These two proteins complement each other to form dCas9-GFP14xGFP, allowing GFP fluorescence restoration. Upon transfecting this cell line with a rcccDNA-CRISPR tag and a CRISPR tag targeting sgRNA, dCas9-GFP14xGFP complex binds to the rcccDNA-CRISPR tag guided by the sgRNA. A single copy of rcccDNA could bind up to 252 complemented GFP molecules, detectable as a spot using fluorescence microscopy [118].

Interestingly, the CRISPR tag is flanked by splice donors and acceptors, so post-transcriptional splicing generates mature HBV RNAs devoid of this tag, thereby preserving viral antigens’ expression and replication. By performing the colabeling of rcccDNA with transcription sites (TSs) via RNA FISH, the authors demonstrated that, at low to medium copy numbers (<20 foci), the colocalization of TSs with CRISPR foci was near-perfect. Furthermore, the histone PTMs (H4ac, H3ac, H3K9me3, H3K4me3, and H3K27me3) of rcccDNA were comparable with those of bona fide cccDNA formed upon infection and were not altered during the imaging process (details of cccDNA histone PTMs are provided in Section 2.2). This study successfully recapitulated the key aspects of cccDNA biology regulation, such as accessibility via HBx (Section 2.3) and the cell proliferation-induced loss of rcccDNA with a random distribution to daughter cells (as mentioned in the introduction section). Altogether, this CRISPR tag system could be instrumental in understanding the transcriptional and epigenetic regulation of cccDNA, as well as the dynamics and fate of cccDNA in real time at the single-cell level. However, this system has a limitation: it cannot be used to detect de novo established cccDNA formed via the nuclear recycling of rcDNA through fluorescence microscopy, as the CRISPR tag is spliced out from mature pgRNA [118].

Seeger developed a CRISPR-based reporter system to investigate HBV cccDNA biology [119] by adapting a system originally created by Moller et al. [120]. This system produces extrachromosomal circular (ec) DNA from a plasmid integrated into the host genome. To mimic cccDNA, Seeger included HBV DNA sequences encoding wild-type or mutant HBx proteins on ecDNA [119]. Similar to the findings by Ding et al. [118], key aspects of cccDNA biology were recapitulated, such as HBx enhancing eGFP-cccDNA expression or reactivating silenced eGFP-cccDNA expression, restricting GFP expression via the SMC5/6 complex, and its loss during cell division.

These new tools provide opportunities to study the fate of HBV cccDNA during cell division, and they are valuable in testing drugs that target cellular factors involved in cccDNA transcription or maintenance.

## 8. Conclusions and Perspectives

CRISPR/Cas has revolutionized both basic and translational research and has been extensively explored as a tool against various infectious viruses [43,44]. As summarized in this review, novel CRISPR-based tools have proven to be efficient in investigating HBV–host interactions, deciphering cccDNA biology, and developing innovative antiviral approaches and HBV-diagnostic platforms. However, challenges associated with CRISPR/Cas therapy, particularly with the prototype Cas9, such as off-target effects, cellular toxicity, delivery methods, viral escape due to genomic heterogeneity, immunogenicity, etc., must be carefully considered [121]. A comprehensive analysis of the off-targets using whole-genome unbiased approaches (such as DISCOVER—Seq, GUIDE—Seq) is crucial to understanding the consequence of gene editing and ensuring its safety [70].

Regarding gRNA design, a significant challenge is low on-target editing due to the rapid degradation of gRNAs via cellular nucleases. This issue can be mitigated by introducing chemical modifications in gRNA, which improve their stability [70,122]. It would be advantageous to design gRNAs that can inhibit the expression of both cccDNA and integrated HBV DNA sequences to suppress viral persistence. However, gRNA design should be performed with caution, as integration can impact the development of novel CRISPR-based therapeutics [11].

The method used for gene delivery also requires careful consideration. LNPs are a clinically relevant vehicle for delivering gene-editor mRNA, and they have been evaluated against liver-related diseases: e.g., to deliver Cas9 nuclease encoding mRNA in order to treat transthyretin amyloidosis ([94] and NCT06128629 Phase 3) and to deliver base editor-encoding mRNA (NCT06389877 Phase 1/2 for patients with alpha-1 antitrypsin deficiency and NCT06164730 Phase 1 for patients with heterozygous familial hypercholesterolemia). Interestingly, this latter base editing human trial aims at permanently switching off PCSK9, a gene highly expressed in the liver [96], offering hope that base editors could be used in clinics to treat other liver-related diseases such as CHB (Table 1). LNPs were also shown to achieve the high transfection of several liver cell types, such as hepatocytes, Kupffer cells, and endothelial cells [123], and they have been explored by several research groups for delivering CRISPR/Cas9 components to target HBV DNA (Table 2). Other delivery nanoplatforms, such as functionalized gold nanoparticles, polyethylenimine-based magnetic nanoparticles, and DNA nanocarriers, could be tested as alternative non-viral methods of delivery [124].

In the absence of therapy, when HBsAg clearance persists with no detectable virus and normal liver function, the patient is considered to have achieved a “functional cure”, which is the current goal for emerging therapies. Programmable gene editing tools, such as base editing, could be promising for achieving this goal. Since hepatitis D virus requires HBsAg for its propagation and entry into hepatocytes [125], such a therapeutic approach may also be applied against HBV/HDV coinfections.

Unlike siRNAs or ASOs, which target HBV RNAs [126] and may require several administrations to prevent viral relapse, base editing directly mutates HBV genomic reservoirs, offering the advantage of preventing viral relapse from the edited molecule, potentially with a single or a few administrations.

Delivery efficiency will be central to ensuring that base editing mutates enough HBV DNA molecules to allow the immune system control of residual HBV replication. Notably, traces of serum HBV DNA can be detected in patients with a resolved acute HBV infection, indicating that, while cccDNA may persist in the liver, HBV infection is immune-controlled [127]. Optimizing strategies to improve editing efficiency, such as multiple dosing of the gene editor [128] or combining treatment with the standard-of-care NAs to suppress HBV replication and block de novo reinfection, may be necessary. Designing a gene editing-based functional cure for CHB patients will require further in vivo studies in models supporting long-term HBV replication and reinfection to assess the risk of an HBV rebound [13,15,16].

Additionally, RNA-targeting endonuclease, Cas13 displays distinct advantages over other RNA-targeting approaches [129,130]. For instance, CRISPR/Cas13 can be engineered to target specific RNA sequences with high precision and reduced off-targets. Further, Cas13 directly cleaves target RNA, whereas siRNAs rely on RNA-induced silencing complex (RISC) to induce RNA degradation, and ASOs typically interact with RNA and promote degradation via RNase H, which may not always be as effective as the direct cleavage mechanism employed by Cas13.

Driven by the need for precise and highly versatile gene editing technology, prime editing is another promising method for gene manipulation. Prime editing allows DNA substitutions, small insertions, and deletions to be introduced at targeted sites without directly creating DSBs [131], and it could be explored in the future to target HBV DNA.

For HBV diagnosis, CRISPR-based diagnostics could be a boom, especially for samples with low viral copy numbers, such as in occult HBV infection [132], in which the detection of viral replication evades standard tests and fails to detect viral replication, leading to misdiagnosis, and the risk of viral reactivation with immunosuppression. With correct diagnosis, these cases could be identified and monitored more effectively. However, challenges may arise when CRISPR-based therapeutics are used alongside CRISPR-based diagnostics to follow up with patients who would have undergone CRISPR-based therapy. For instance, diagnostic results could be misinterpreted due to issues such as the reduced accessibility Cas9 to transcriptionally inactive cccDNA [133] and the inhibition of binding of gRNAs due to mutations at the target site introduced by CRISPR/Cas therapy.

In summary, gene editing tools hold significant promise for inactivating HBV genomes. However, combining gene editing with other antiviral agents targeting different steps of the HBV life cycle or with immunotherapeutic agents (such as checkpoint inhibitors or a therapeutic vaccine) to boost the host immune system appears essential. Determining the optimal sequence for administering gene editors, immune modulators, and antivirals remains a significant scientific challenge, and it needs extensive (pre)-clinical trials [134].

## Figures and Tables

**Table 1:** Recent approaches used for liver-targeted delivery of CRISPR/Cas9.

Delivery Vehicle	Cargo	Advantages	Limitations	Examples	References
Adeno-associated viruses (AAVs)	DNA-encoding Cas9/sgRNA	Mainly episomal and, hence, minimal risk of integration into the host genome, low inflammatory response, high transduction efficiency, pseudotyping possible	Limited cargo packaging capacity, serotype-dependent preexisting immunity, long-term expression of editing components, off-target risk	Liver function restoration in mouse model of Crigler–Najjar syndrome for the insertion of therapeutic cDNA into the albumin locus	Maestro et al., 2021; De Caneva et al., 2019 [55,92]
Nanoblades	Engineered virus-like particles loaded with Cas9-sgRNA RNPs	Non-viral, can be used for rapid and transient expression, minimal off-targets, can be complexed with other components, such as DNA repair templates	Lower editing, need further improvements	Editing of *Hpd* gene in NRG mice	Mangeot et al., 2019 [93]
LNPs	Gene editor-encoding mRNA/sgRNA	Non-viral, low immunogenicity, no integration risk, can be used for rapid and transient expression, low toxicity, flexible cargo packaging capacity	May have lower transduction efficiency, limited targeting without modification	Cas9 nuclease-based editing of *TTR* gene to treat transthyretin amyloidosis in patients Base editing of the PiZ gene to treat alpha-1 antitrypsin deficiency in PiZ-transgenic mice Base editing of *PCSK9* gene to treat familial hypercholesterolemia in patients	Gillmore et al., 2021; Packer et al., 2022; Horie and Ono, 2024 [94,95,96]
Engineered VLPs (eVLPs)	Engineered virus-like particles loaded with base editor-sgRNA RNPs	Non-viral, can be used for rapid and transient expression, minimal off-targets	Needs more characterization, including contents that may be packaged and pharmacokinetics	Base editing of *PCSK9* gene in mice	Banskota et al., 2022 [97]

**Table 2:** Newer approaches to the liver-targeted delivery of CRISPR/Cas9 to target HBV.

Delivery Vehicle	Cargo	In Vivo Model System	References
LNPs	Cas9-encoding mRNA and HBV-targeting gRNA	AAV-HBV1.04 mice, 1.28-mer (gt A) HBV-integrated genome mice, HBV-infected tree shrews	Yi et al., 2023 [68]
LNPs	BE-encoding mRNA and HBV-targeting gRNA	HBV minicircle mouse	Smekalova and Martinez et al., 2024 [77]
LNPs	EE-encoding mRNA and HBV-targeting gRNA	AAV-HBV, HBV transgenic, HBV-infected FRG chimeric mice	Yesseinia 2023 and 2024; Brian, 2023 [83,84,85]
EVs	RNP containing Cas9 and HBV-targeting gRNA	1.2x HBV replicon (gt C)-replicating mice and HBV (gt A) transgenic mice	Zeng et al., 2024 [65]

**Table 3:** Comparison between different HBV detection assays.

Assays	Target Molecule	Advantages	Limitations	LOD
ELISA	HBV antigen or antibody	Simple, cost-effective, high throughput	Lower sensitivity	0.1–0.5 ng/mL
qPCR	HBV DNA	High sensitivity and specificity, allows quantitative monitoring of viral load	Requires specialized equipment and technical expertise, expensive	10–100 IU/mL (approx. 50–500 copies/mL)
Nucleic acid sequencing	HBV DNA	Provides detailed viral genetic information (genotypes and mutations)	High cost, needs advanced equipment and bioinformatics expertise	10–100 copies/mL
CRISPR/Cas	HBV DNA	Ultra-sensitive, potential for high specificity	Still in experimental stages for diagnostic use	1 copy/µL

**Table 4:** CRISPR-based diagnostics for HBV.

Cas Type	Amplification	Detection	LOD	Reference
Cas13a	PCR followed by T7 transcription	Fluorescence	1 copy/test	Wang S et al., 2021 [100]
Cas12a	LAMP	Fluorescence and lateral flow	1 copy/μL	Ding et al., 2021 [101]
Cas12a	-	Surface-enhanced Raman spectroscopy (SERS)	1 aM	Choi et al., 2021 [102]
Cas12b	MCDA	Fluorescence and lateral flow	10 copies/test	Chen et al., 2021 [103]
Cas12a	SDA	Colorimetric	41.8 fM	Gong et al., 2021 [104]
Cas13a	RCA⟶PCR⟶T7 transcription	Fluorescence	1 copy/μL	Zhang et al., 2022 [105]
Cas12a	-	Colorimetric	0.5 pM	Tao et al., 2022 [106]
Cas12a	PCR	Nanopore sensing	5 aM	Wang et al., 2022 [107]
Cas12a	-	Surface-enhanced Raman spectroscopy (SERS)	100 fM	Du et al., 2023 [108]
Cas12a	RCA	Fluorescence	1.502 pM	Liu et al., 2023 [109]
Cas12a	Entropy-driven 3D DNA walking machine	Electrochemiluminescence	17 aM	Li et al., 2023 [110]
Cas13a	RAA⟶T7 transcription	Fluorescence and lateral flow assay (LFA)	10 copy/μL	Tian et al., 2023 [111]
Cas12a	SDA	Fluorescence/PGM/LFA	55.1 fM	Li et al., 2024 [112]
Cas12b	LAMP and fluorescence detection-one step detection.	Fluorescence	25 copies/mL	Xu et al., 2024 [113]

## Data Availability

Not applicable.

## Abbreviations

HBV, hepatitis B virus; CRISPR, Clustered Regularly Interspaced Short Palindromic Repeats; cccDNA, covalently closed circular DNA; rcDNA, relaxed circular DNA; HCC, hepatocellular carcinoma; CHB, chronic hepatitis B; NTCP, sodium taurocholate co-transporting polypeptide; SL10A1 (solute carrier family 10 member 1); DSB, double-strand break; SVPs, subviral particles; dsDNA, double-strand DNA; HBsAg, hepatitis B surface antigen; S-HBsAg, small HBsAg; M-HBsAg, middle HBsAg; L-HBsAg, large HBsAg; HBc, hepatitis core protein; pgRNA, pre-genomic RNA; Pol, HBV polymerase; RT, reverse transcriptase; HBx, hepatitis B virus X protein; CpG, cytosine–phosphate–guanine; 5mC, 5-methyl cytosine; H3, histone 3; H4, histone 4; ChIP, chromatin immunoprecipitation; HDAC, histone deacetyltransferase; SETDB1, SET domain bifurcated 1; HMGB1, high-mobility group box-1; SMC5/6, structural maintenance of chromosomes complex 5/6; SLF2, SMC5-SMC6 localization factor 2; NA, nucleos(t)ide analogue; PAM, protospacer adjacent motif; gRNA, guide RNA; PHHs, primary human hepatocytes; EVs, extracellular vesicles; LNPs, lipid nanoparticles; ZEB, zinc finger E-box binding homeobox; rcccDNA, recombinant cccDNA; EMT, epithelial–mesenchymal transition; RNP, ribonucleoprotein complex; dpi, days post-infection; dpn, days post-nucleofection; CRY2; cryptochrome 2: AAV, adenoassociated virus; EEs, epigenetic editors; dCas9, dead Cas9; nCas9, nickase Cas9; CRISPRa, CRISPR activation; CRISPRi, CRISPR interference; LAMP, loop assisted isothermal amplification; SDA, strand displacement amplification; MCDA, multiple cross displacement amplification; RAA, recombinase-aided amplification; LOD, limit of detection; 3D, three-dimensional; HOLMES, one-hour, low-cost, multipurpose, highly efficient system; SERS, surface-enhanced Raman spectroscopy; TS, transcription sites; PCSK9, proprotein convertase subtilisin/kexin type 9; siRNAs, small interfering RNAs; ASOs, antisense oligonucleotides; DISCOVER-seq, discovery of in situ Cas off-targets and verification by sequencing; GUIDE Seq, genome-wide, unbiased identification of double-stranded end sequencing; HDV, hepatitis D virus; AAV, adeno-associated virus; TTR, transthyretin-encoding gene; hpd, hydroxyphenylpyruvate dioxygenase; eVLPs, engineered virus-like particles.
